# Effect of Combination Antibiotic Empirical Therapy on Mortality in Neutropenic Cancer Patients with *Pseudomonas aeruginosa* Pneumonia

**DOI:** 10.3390/microorganisms10040733

**Published:** 2022-03-29

**Authors:** Adaia Albasanz-Puig, Xavier Durà-Miralles, Júlia Laporte-Amargós, Alberto Mussetti, Isabel Ruiz-Camps, Pedro Puerta-Alcalde, Edson Abdala, Chiara Oltolini, Murat Akova, José Miguel Montejo, Malgorzata Mikulska, Pilar Martín-Dávila, Fabián Herrera, Oriol Gasch, Lubos Drgona, Hugo Manuel Paz Morales, Anne-Sophie Brunel, Estefanía García, Burcu Isler, Winfried V. Kern, Pilar Retamar-Gentil, José María Aguado, Milagros Montero, Souha S. Kanj, Oguz R. Sipahi, Sebnem Calik, Ignacio Márquez-Gómez, Jorge I. Marin, Marisa Z. R. Gomes, Philipp Hemmati, Rafael Araos, Maddalena Peghin, José Luis del Pozo, Lucrecia Yáñez, Robert Tilley, Adriana Manzur, Andres Novo, Natàlia Pallarès, Alba Bergas, Jordi Carratalà, Carlota Gudiol, on behalf of the IRONIC Study Group

**Affiliations:** 1 Infectious Diseases Department, Bellvitge University Hospital, IDIBELL, University of Barcelona, 08907 Barcelona, Spain; adaia.albasanz@gmail.com (A.A.-P.); xavierduramiralles@gmail.com (X.D.-M.); jlaporte@iconcologia.net (J.L.-A.); albabergas@gmail.com (A.B.); cgudiol@iconcologia.net (C.G.); 2 Centro de Investigación Biomédica en Red de Enfermedades Infecciosas (CIBERINFEC), Instituto de Salud Carlos III, 28029 Madrid, Spain; iruiz@vhebron.net (I.R.-C.); pmartindav@gmail.com (P.M.-D.); pilaretamar@hotmail.com (P.R.-G.); jaguadog1@gmail.com (J.M.A.); 3 Hematology Department, Catalan Institute of Oncology (ICO)-Duran i Reynals Hospital, IDIBELL, 08907 Barcelona, Spain; amussetti@iconcologia.net; 4 Infectious Diseases Department, Vall d’Hebron University Hospital, 08035 Barcelona, Spain; 5 Infectious Diseases Department, Hospital Clínic i Provincial, 08035 Barcelona, Spain; puerta@clinic.cat; 6 Instituto do Câncer do Estado de São Paulo, Faculty of Medicine, University of São Paulo, Sao Paulo 01246, Brazil; eabdala@uol.com.br; 7 Unit of Infectious and Tropical Diseases, IRCCS San Raffaele Scientific Institute, 20132 Milan, Italy; oltolini.chiara@hsr.it; 8 Department of Infectious Diseases, Hacettepe University School of Medicine, 06230 Ankara, Turkey; akova.murat@gmail.com; 9 Infectious Diseases Department, Cruces University Hospital, 48903 Bilbao, Spain; josemiguel.montejobaranda@osakidetza.eus; 10 Division of Infectious Diseases, Department of Health Sciences, University of Genoa (DISSAL) and Ospedale Policlinico San Martino, 16132 Genoa, Italy; m.mikulska@unige.it; 11 Infectious Diseases Department, Ramon y Cajal Hospital, 28034 Madrid, Spain; 12 Infectious Diseases Section, Department of Medicine, Centro de Educación Médica e Investigaciones Clínicas (CEMIC), Buenos Aires C1430EFA, Argentina; fabian1961@gmail.com; 13 Infectious Diseases Department, Parc Taulí University Hospital, Sabadell, 08208 Barcelona, Spain; ogasch@tauli.cat; 14 Oncohematology Department, Comenius University and National Cancer Institute, 81499 Bratislava, Slovakia; lubos.drgona@gmail.com; 15 Infectious Diseases Department, Hospital Erasto Gaertner, Curitiba 81520-060, Brazil; moraleshmp@gmail.com; 16 Infectious Diseases Department, Department of Medicine, Lausanne University Hospital, Centre Hospitalier Universitaire Vaudois (CHUV), 1011 Lausanne, Switzerland; anne-sophie.brunel@chuv.ch; 17 Haematology Department, Reina Sofía University Hospital-IMIBIC-UCO, 14004 Córdoba, Spain; estefania.garcia.sspa@juntadeandalucia.es; 18 Department of Infectious Diseases and Clinical Microbiology, Istanbul Education and Research Hospital, Istanbul 34668, Turkey; burcubayrak85@gmail.com; 19 Division of Infectious Diseases, Department of Medicine II, University of Freiburg Medical Center and Faculty of Medicine, 79110 Freiburg, Germany; winfried.kern@uniklinik-freiburg.de; 20 Unit of Infectious Diseases and Microbiology, University Hospital Virgen Macarena, Department of Medicine, Universidad de Sevilla, Institute of Biomedicine of Seville, Virgen del Rocío and Virgen Macarena University Hospitals/CSIC/University of Seville, 41013 Seville, Spain; 21 Infectious Diseases Unit, Instituto de Investigación Hospital “12 de Octubre” (i + 12), “12 de Octubre” University Hospital, School of Medicine, Universidad Complutense, 28041 Madrid, Spain; 22 Infectious Diseases Service, Hospital del Mar, Infectious Pathology and Antimicrobials Research Group (IPAR), Institut Hospital del Mar d’Investigations Mèdiques (IMIM), Universitat Autònoma de Barcelona (UAB), CEXS-Universitat Pompeu Fabra, 08003 Barcelona, Spain; mmontero@parcdesalutmar.cat; 23 Infectious Diseases Division, American University of Beirut Medical Center, Beirut 110236, Lebanon; sk11@aub.edu.lb; 24 Faculty of Medicine, Ege University, Izmir 35040, Turkey; oguz.resat.sipahi@gmail.com; 25 Department of Infectious Diseases and Clinical Microbiology, University of Health Science Izmir Bozyaka Training and Research Hospital, Izmir 35170, Turkey; sebnemozkoren@yahoo.com; 26 Infectious Diseases Department, Hospital Regional de Málaga, 29010 Málaga, Spain; nachomarquezgomez@gmail.com; 27 Infectious Diseases and Clinical Microbiology Department, Clínica Maraya, Pereira, Colombia, Critical Care and Clinical Microbiology Department, Manizales 170001-17, Colombia; jimarin.uribe@gmail.com; 28 Hospital Federal dos Servidores do Estado, Instituto Oswaldo Cruz, Fundação Oswaldo Cruz, Ministério da Saúde, Rio de Janeiro 20221-161, Brazil; marisargomes@ioc.fiocruz.br; 29 Department of Hematology, Oncology and Palliative Care, Klinikum Ernst von Bergmann, Academic Teaching Hospital of Charité University Medical School, 10117 Berlin, Germany; philipp@hemmati.de; 30 Facultad de Medicina Clínica, Instituto de Ciencias e Innovación en Medicina, Alemana Universidad del Desarrollo, Santiago de Chile, and Millennium Initiative for Collaborative Research on Bacterial Resistance (MICROB-R), Santiago de Chile 12461, Chile; rafaaraos@gmail.com; 31 Infectious Diseases Clinic, Department of Medicine, University of Udine and Azienda Sanitaria Universitaria Integrata, 33100 Udine, Italy; maddalena.peghin@gmail.com; 32 Infectious Diseases and Microbiology Unit, Faculty of Medicine, Navarra University Clinic, 31008 Pamplona, Spain; jdelpozo@unav.es; 33 Haematology Department, Marqués de Valdecilla University Hospital, 39008 Santander, Spain; lucrecia.yanez@scsalud.es; 34 Microbiology Department, University Hospitals Plymouth NHS Trust, Plymouth 8DH, UK; robert.tilley@nhs.net; 35 Infectious Diseases, Hospital Rawson, San Juan J5400, Argentina; admanzur74@yahoo.com.ar; 36 Haematology Department, Son Espases University Hospital, 07120 Palma de Mallorca, Spain; andres.novo@ssib.es; 37 Statistics Advisory Service, Institute of Biomedical Research of Bellvitge, Rovira i Virgili University, 08908 Tarragona, Spain; npallares@idibell.cat; 38 Infectious Diseases Unit, Catalan Institute of Oncology (ICO), Duran i Reynals Hospital, IDIBELL, 08908 Barcelona, Spain

**Keywords:** *Pseudomonas aeruginosa*, bloodstream infection, pneumonia, septic shock, neutropenia

## Abstract

To assess the effect of combination antibiotic empirical therapy on 30-day case-fatality rate in neutropenic cancer patients with *Pseudomonas aeruginosa* (PA) bacteremic pneumonia. This was a multinational, retrospective cohort study of neutropenic onco-hematological patients with PA bloodstream infection (BSI) (2006–2018). The effect of appropriate empirical combination therapy, appropriate monotherapy and inappropriate empirical antibiotic therapy [IEAT] on 30-day case-fatality was assessed only in patients with PA bacteremic pneumonia. Among 1017 PA BSI episodes, pneumonia was the source of BSI in 294 (28.9%). Among those, 52 (17.7%) were caused by a multidrug-resistant (MDR) strain and 68 (23.1%) received IEAT, mainly when the infection was caused by an MDR strain [38/52 (73.1%) vs. 30/242 (12.4%); *p* < 0.001]. The 30-day case-fatality rate was higher in patients with PA bacteremic pneumonia than in those with PA BSI from other sources (55.1% vs. 31.4%; *p* < 0.001). IEAT was associated with increased 30-day case-fatality (aHR 1.44 [95%CI 1.01–2.03]; *p* = 0.042), whereas the use of appropriate combination empirical treatment was independently associated with improved survival (aHR 0.46 [95%CI 0.27–0.78]; *p* = 0.004). Appropriate empirical monotherapy was not associated with improved overall survival (aHR 1.25 [95%CI 0.76–2.05]; *p* = 0.39). Combination antibiotic empirical therapy should be administered promptly in febrile neutropenic patients with suspected pneumonia as the source of infection.

## 1. Introduction

*Pseudomonas aeruginosa* (PA) bloodstream infection (BSI) remains one of the leading causes of death among neutropenic cancer patients [1,2]. The administration of inadequate initial empirical antibiotic treatment for PA BSI in this population has been associated with impaired outcomes [3,4,5]. Importantly, due to the worrisome increase in multidrug resistance among PA isolates, the rates of inadequate empirical antibiotic treatment are increasing with a detrimental impact on mortality [1,6].

Data from a few retrospective studies suggest that an initial combination empirical therapy with two active antipseudomonal antibiotics raises levels of appropriate treatment in patients with PA BSI, and consequently improves outcomes [4,7]. Nevertheless, the possible benefit of combined treatment over monotherapy is still a matter of vivid debate [8,9,10,11]. The issue is controversial because the mortality attributed to PA BSI might not be determined only by the appropriateness of the empirical antibiotic treatment, but also by the source of infection, the baseline characteristics of the infected host and the severity of the clinical presentation [12,13,14]. In this regard, bacterial pneumonia is common in febrile neutropenic patients, with a prevalence that ranges from 16% to 40% of cases [15]. More specifically, 31.5% of patients with acute leukemia [16] and 64% of allogeneic hematopoietic stem cell transplant recipients will develop an episode of pneumonia [17], with unacceptably high case-fatality rates, particularly when compared with infections from other sources [18,19,20].

PA is one of the main causative agents of pneumonia in neutropenic cancer patients [21,22]. It frequently presents with BSI, septic shock and/or requires intensive care unit admission [20,23,24]. Even though the most recent Infectious Diseases Society of America/American Thoracic Society (IDSA/ATS) guidelines recommend an initial empirical therapy with two active antipseudomonal antibiotics for hospital-acquired pneumonia in patients at high risk for mortality and/or antimicrobial resistance [25], the true impact of this strategy in febrile neutropenic patients with PA pneumonia is still unknown. In fact, the currently available guidelines for the management of febrile neutropenia lack specific recommendations regarding the empirical treatment of pneumonia in neutropenic patients [26].

In this study, we assess the effect of combination antibiotic empirical therapy versus monotherapy on 30-day mortality in neutropenic cancer patients with PA bacteremic pneumonia.

## 2. Materials and Methods

### 2.1. Study Design and Setting

This study is part of the IRONIC project: a multicenter, international, retrospective cohort study conducted from 1 January 2006 to 31 May 2018 at 34 centers in 12 countries. The number of participating centers is provided in the Supplementary Material. The number of patients recruited at each participating center has been reported elsewhere [27].

### 2.2. Ethics

The study was approved by the Institutional Review Board at Bellvitge University Hospital (local reference number PR408/17) and by the local Research Ethics Committees at the participating centers. It was conducted in accordance with the Declaration of Helsinki guidelines. The need for informed consent was waived by the Clinical Research Ethics Committee due to the retrospective design. The study results are reported following the STROBE recommendations [28] (Supplementary Material).

### 2.3. Participants

All adult (≥18 years) onco-hematological neutropenic patients, including hematopoietic stem cell transplant (HSCT) recipients, were eligible for the study if they were diagnosed with at least one episode of PA BSI during the study period. Subsequent episodes caused by PA occurring in the same patient were included in the study if they occurred at intervals of >1 month. Only monomicrobial PA BSI episodes were selected for this study. The exclusion criteria were unavailability of key data (related to death) and polymicrobial BSI. The follow-up period was 30 days from BSI onset.

### 2.4. Variables

Data regarding baseline characteristics, clinical and microbiological features and endpoints were collected. Empirical antibiotic therapy was considered when the antibiotic was administered before reception of definitive susceptibility results. Appropriate empirical antibiotic therapy was defined when patients received at least one in vitro active antibiotic against the PA strain. Empirical combination therapy was considered appropriate when both antibiotics that were empirically administered were active against the PA strain. If a patient received two empirical antibiotics but only one was active, this was considered appropriate monotherapy. Inappropriate empirical antibiotic treatment (IEAT) was considered when the patient did not receive any empirical antibiotic with in vitro activity or an empirical antibiotic therapy was lacking. In addition, in the cohort of patients with PA pneumonia, empirical monotherapy with an aminoglycoside was considered inappropriate. Thus, three treatment categories were observed: appropriate empirical combination therapy, appropriate empirical monotherapy and IEAT. The antipseudomonal β-lactams were uniformly administered at the current standard doses for the treatment of febrile neutropenia [26,29]. In case of renal impairment, the dosing was adjusted accordingly.

### 2.5. Outcomes

The 30-day case-fatality rate of patients with bacteremic PA pneumonia was compared with those with BSI from other sources. The rate of persistent BSI, need for intensive care unit (ICU) admission and mechanical ventilation were also analyzed. Risk factors for overall 30-day case-fatality and the effect of the use of appropriate combination antibiotic empirical therapy, monotherapy or IEAT on mortality were analyzed only in patients with PA pneumonia.

### 2.6. Microbiological Studies

Clinical samples were processed at the microbiology laboratories of each participating center in accordance with standard operating procedures. PA was identified using standard microbiological techniques at each center. In vitro susceptibility was determined according to the EUCAST recommendations [30], except at the Lebanese center and at one center from Argentina where the CLSI breakpoints were used, and in the center in the UK where the BSAC recommendations were used before 2016 [31]. PA isolate phenotypes were stratified in accordance with recent standard definitions [32].

### 2.7. Definitions

Neutropenia and severe neutropenia were defined as an absolute neutrophil count below 0.5 × 10^9^ cells/mm and 0.1 × 10^9^ cells/mm, respectively. Previous corticosteroid treatment was defined as the administration of ≥20 mg of prednisone, or equivalent dosing, for at least four weeks within 30 days of BSI onset. Patients with refractory/relapsed disease and those receiving palliative chemotherapy were classified as having refractory disease.

Bacteremic PA pneumonia was defined as the presence of an acute respiratory illness and a new pulmonary infiltrate on a chest radiograph and/or CT in association with concurrent PA BSI. Other BSI sources were established using standard US Centers for Disease Control and Prevention criteria for secondary BSI [33]. In addition, the source of BSI was defined as unknown or endogenous in patients in whom no other sources were identified. Septic shock was defined as a systolic blood pressure <90 mmHg that was unresponsive to fluid treatment or required vasoactive drug therapy [34]. Mucositis was considered in patients with ulcerative lesions involving only the oral cavity. Comorbidities were defined as the presence of one or more of the following diseases: chronic obstructive pulmonary disease, heart disease, hepatic disease, diabetes mellitus, renal failure and cerebrovascular disease. Persistent BSI was considered if blood cultures were positive after 48 h of adequate antibiotic therapy. The 30-day case-fatality rate was defined as death from any cause within 30 days of BSI onset.

### 2.8. Statistical Analysis

To define cohort characteristics, categorical variables were presented as the number of cases and percentages, while continuous variables were presented as the mean and standard deviation (SD) or median and interquartile range (IQR). Continuous variables were compared using the Student’s *t*-test or the Mann–Whitney U test where appropriate. Fisher’s exact test or Pearson’s χ2 test were applied to assess the relationship between categorical variables.

Time until death, or last follow-up, was assessed using the Kaplan–Meier estimator. The log-rank test was used to compare the survival curves between treatment strategies. The Cox proportional hazards model was used to perform an adjusted analysis of the treatment strategies with age, sex, septic shock and the presence of multidrug resistance as clinically relevant factors. The proportionality of risks in the Cox model was checked using the Schoenfeld residuals. The presence of septic shock at admission violated the proportional hazard assumption of the Cox model. To address this problem, we performed a time-dependent covariates analysis on septic shock. Data were split into three groups (from day 0 to day 2, from day 2 to day 10, and from day 10 to day 30), defined after the exploration of the Schoenfeld residuals plot, to estimate different coefficients over different time intervals [35]. The effects of the treatment strategies were reported with hazard ratio (HR) and 95% confidence interval adjusted for age, sex, and multi-drug resistant pneumonia. Statistical analyses were performed with R software 4.1.0.

## 3. Results

### 3.1. Clinical Characteristics

Among the 1217 eligible PA BSI episodes, pneumonia was the second most frequent source, with 311 episodes (25.5%), after an endogenous source (37.4%). Two hundred episodes met at least one of the exclusion criteria and were excluded from the analysis, leaving 1017 included episodes, in which pneumonia was the source of BSI in 294 (28.9%). Baseline characteristics of patients included in the study are detailed in the supplementary material (Table S1). Patients with bacteremic PA pneumonia had more comorbidities and presented more frequently with septic shock than those with PA BSI from other sources. In total, 17.7% (52/294) of PA pneumonia episodes were caused by multidrug-resistant (MDR) strains, and 30.6% (90/294) were due to carbapenem-resistant isolates, without significant differences between groups.

### 3.2. Treatment Characteristics

Table 1 shows the different treatment regimens administered in patients with PA pneumonia. Overall, an antipseudomonal β-lactam in monotherapy was the most frequent empirical antibiotic used (173/294, 58.9%). When a combination empirical therapy was preferred, a combination of a β-lactam plus an aminoglycoside was predominant (87/118, 73.7%).

Of note, 68 episodes (23.1%) received IEAT, particularly when the PA BSI was caused by an MDR strain [38/52 (73.1%) vs. 30/242 (12.4%); *p* < 0.001]. Almost 49% of these 68 episodes were initially treated with an antipseudomonal β-lactam in monotherapy with no in vitro activity against the PA isolate (mainly carbapenems or piperacilin/tazobactam). Compared with PA BSI from other sources, no differences were found between the rate of inappropriate empirical antibiotic treatment [68/294 (23.1%) vs. 142/723 (19.6%); *p* = 0.29]. Among those episodes caused by a MDR PA strain, only 14 received an appropriate empirical antibiotic treatment that was mainly based on a monotherapy (10 of 14 cases).

### 3.3. Outcomes

The 30-day case-fatality rate was significantly higher in patients with PA bacteremic pneumonia than in those with PA BSI from other sources (Table 2). Moreover, the rates of persistent BSI and the need for ICU admission and mechanical ventilation were also higher in patients with pneumonia.

### 3.4. Risk Factors for Mortality in Patients with Bacteremic PA Pneumonia

In the univariate Cox analysis, septic shock at presentation (HR 3.56 [95%CI 2.56–4.94]; *p* < 0.001), infection due to a MDR strain (HR 2.05 [95%CI 1.43–2.93]; *p* < 0.001) and IEAT (HR 1.57 [95%CI 1.21–2.21]; *p* = 0.009) were associated with higher 30-day case-fatality rate, whereas the use of an appropriate empirical combination treatment was associated with improved survival (HR 0.46 [95%CI 0.29–0.72]; *p* = 0.001). In the multivariate Cox analysis, receiving IEAT was an independent risk factor for increased 30-day case-fatality (aHR 1.44 [95%CI 1.01–2.03]; *p* = 0.042).

**Figure 1:**
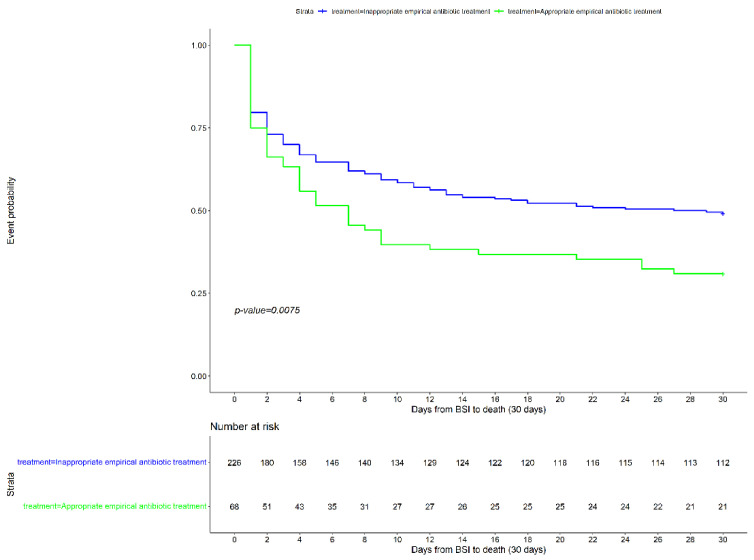
Kaplan–Meier survival analysis of patients with *Pseudomonas aeruginosa* bacteremic pneumonia, stratified by the appropriateness of empirical treatment.

**Figure 2:**
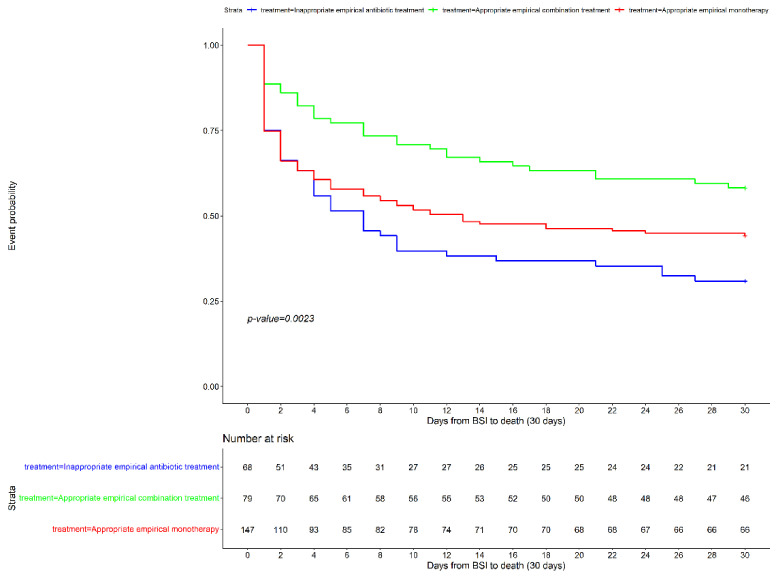
Kaplan–Meier survival analysis of patients with *Pseudomonas aeruginosa* bacteremic pneumonia, stratified by treatment group.

In addition, when focusing on the impact of the three treatment strategies, we observed that the benefit on survival of the administration of an appropriate empirical combination treatment was maintained after adjusting for potential confounders (aHR 0.46 [95%CI 0.27–0.78]; *p* = 0.004). The univariate and multivariate Cox models are detailed in Table 3 and Table 4). Figure 1 and Figure 2 show the Kaplan–Meier curves stratified by the appropriateness of empirical treatment.

## 4. Discussion

In this large multicenter, international cohort study of high-risk neutropenic cancer patients, we found that pneumonia was a frequent cause of PA BSI and was associated with a strikingly high 30-day case-fatality rate. Among patients with bacteremic PA pneumonia, the presence of septic shock and the use of an IEAT had a negative impact on overall survival. Conversely, the administration of appropriate initial combined empirical antibiotic treatment, mainly based on an active antipseudomonal β-lactam and an aminoglycoside, was independently associated with improved outcomes.

We found that 23% of patients with bacteremic PA pneumonia received IEAT, a percentage that rose significantly in the case of MDR strains. IEAT was also found to be an independent risk factor for mortality; indeed, it has previously been shown to have a detrimental effect on mortality in neutropenic cancer patients with PA BSI [3,4,6,36] and bacteremic PA pneumonia [7,14]. However, the presence of multidrug resistance was not independently associated with a poor prognosis in our study. In this regard, even though the association between IEAT and multidrug resistance has been previously reported [3,37], its real impact on mortality is still controversial [6,7,12,13,38].

In the current era of emerging antibiotic resistance, it seems reasonable to opt for initial empirical combination treatment in order to increase the probability of adequateness and also to take advantage of the potential antibiotics’ synergism [39]. Nevertheless, the benefit for survival of using a combined empirical antibiotic therapy rather than monotherapy to treat PA severe infections in the general population remains a long-standing controversy [8,37,40]. Specifically, when focusing on PA bacteremic pneumonia, Park et al. found that the use of adequate combined empirical antibiotic treatment was associated with improved 28-day survival and higher 2 and 4-week bacterial eradication rates [7]. Conversely, Recio et al. did not confirm this beneficial effect on mortality [5]; however, these authors found neutropenia to be a risk factor for early 5-day mortality.

Importantly, this knowledge gap is even greater in the setting of febrile neutropenia [41]. Indeed, a meta-analysis by Paul et al. focusing on neutropenic cancer patients found no benefit of combination therapy, although the rate of PA infections was only 1.7% and the number of pneumonia episodes was not specified [11].

Of note, we found that the use of a combined empirical antibiotic treatment for PA bacteremic pneumonia in neutropenic patients, mainly based on a β-lactam and an aminoglycoside, was associated with improved 30-day mortality. Our results may be explained by the synergistic effect of combining an adequate β-lactam antibiotic and aminoglycoside, as suggested by early studies [42]. In this line, in a previous study, we observed an improved early (7-day) mortality rate in those neutropenic cancer patients with GNB BSI who received initial appropriate combination therapy [39]. In addition, the beneficial effect of adding an aminoglycoside to an active β-lactam has recently been shown to be particularly relevant in febrile neutropenic patients presenting with septic shock [43]. Similarly, in our study, the beneficial effect of a combined therapy over monotherapy was mainly observed within the first 7 days of BSI onset, probably when the empirical use of an aminoglycoside is more important to increase the rate of bacterial clearance and consequently improve outcomes.

In this regard, choosing the optimal empirical β-lactam for the treatment of PA pneumonia according to the individual risk of resistance is crucial. Here, the newly available β-lactam + β-lactamase inhibitors may play a very important role [44,45]. The use of colistin instead of an aminoglycoside is an appealing proposal, but no firm conclusions regarding its appropriateness can be drawn from the present study; however, it is an empirical treatment strategy that urgently deserves further investigation.

The main strength of this study is that it is based on one of the largest cohorts of neutropenic cancer patients with PA pneumonia, with a multicenter international design, that allows the generalization of the results. To account for a possible bias, multivariate Cox analyses were performed with septic shock as a time-dependent variable. Nevertheless, this study also has some limitations that should be acknowledged. First, this was not a randomized clinical trial (RCT); thus, the choice of therapy may have been influenced by patient-related variables and by the clinical presentation. Second, we included various empirical combination antibiotic regimens, which may have had different effects on mortality. Finally, some information may have been lost due to the retrospective design, and we may not have adequately controlled for certain confounders.

## 5. Conclusions

In conclusion, we found that appropriate initial combination antibiotic empirical treatment improved 30-day mortality compared with monotherapy in neutropenic patients with PA bacteremic pneumonia. Therefore, combination empirical therapy should be promptly administered in febrile neutropenic patients with suspected pneumonia as the source of infection. However, further RCTs are warranted to confirm our results.

## Figures and Tables

**Table 1:** Treatment characteristics of the 294 patients with *Pseudomonas aeruginosa* bacteremic pneumonia.

**No Antibiotic Treatment (Death < 48 h)**	**3/294 (1)**
**Initial empirical monotherapy**	**173/294 (58.9)**
Piperacillin/tazobactam	84 (48.6)
Antipseudomonal carbapenems (imipenem, meropenem)	54 (31.2)
Antipseudomonal cephalosporins	24 (13.9)
Polymyxins (Colistin/Polymyxin B)	2 (1.1)
Fluoroquinolones	1 (0.6)
Aminoglycoside	1 (0.6)
Others ^a^	7 (4)
**Initial empirical combination therapy**	**118/294 (40.1)**
β-lactam + AG	87 (73.7)
β-lactam + non-AG	27 (22.9)
Non-β-lactam combination	4 (3.4)
**Appropriate empirical treatment**	**226/294 (76.9)**
** Monotherapy ^b^**	**147/226 (65)**
Piperacillin/tazobactam	73 (49.7)
Anti-pseudomonal carbapenems (imipenem, meropenem)	37 (25.2)
Anti-pseudomonal cephalosporins	23 (15.7)
Polymyxins (Colistin, Polymyxin B)	12 (8.2)
Fluoroquinolone (levofloxacin/ciprofloxacin)	2 (1.4)
** Combined therapy**	**79/226 (35)**
β-lactam + AG	59 (74.7)
β-lactam + non-AG	20 (25.3)
**Inappropriate empirical treatment**	**68/294 (23.1)**
** No antibiotic treatment (Death < 48 h)**	**3/68 (4.4)**
** Monotherapy**	**41/68 (60.3)**
Anti-pseudomonal carbapenems (imipenem, meropenem)	16 (39)
Piperacillin/tazobactam	15 (36.6)
Anti-pseudomonal cephalosporins	2 (4.9)
Aminoglycosides	1 (2.4)
Others ^c^	7 (17)
** Combination therapy**	**24/68 (35.3)**
β-lactam + AG ^d^	21 (87.5)
β-lactam + non-AG	1 (4.2)
Non-β-lactam combination	2 (8.3)

Qualitative data are expressed as numbers (%), unless otherwise indicated. Abbreviations: AG: Aminoglycoside. ^a^ Clindamycin (*n* = 1), Azithromycin (*n* = 1), Ceftriaxone (*n* = 2), Amoxicillin/clavulanate (*n* = 2), Metronidazole (*n* = 1). ^b^ 12 patients received an initial empirical combination treatment but were classified as receiving an appropriate empirical monotherapy because only one of the antibiotics showed in vitro activity against the PA strain: Colistin (*n* = 9), fluoroquinolone (*n* = 1), antipseudomonal cephalosporin (*n* = 1), piperacillin/tazobactam (*n* = 1). ^c^ Amoxicillin/clavulanate (*n* = 2), Ceftriaxone (*n* = 2), Metronidazole (*n* = 1), Clindamycin (*n* = 1), Azithromycin (*n* = 1). ^d^ 19 episodes received inappropriate empirical combination antibiotic treatment where only the aminoglycoside showed in vitro activity against the PA isolate.

**Table 2:** Outcomes.

	PA Pneumonia *n* = 294 (%)	PA BSI *n* = 723 (%)	*p*-Value
30-day case-fatality rate	162 (55.1)	227 (31.4)	<0.001
Persistent BSI (48h from BSI onset)	41 (14.5)	71 (9.9)	0.048
ICU admission	126 (42.9)	186 (25.7)	<0.001
Need for mechanical ventilation	83 (28.2)	115 (15.9)	<0.001

Abbreviations: PA: Pseudomonas aeruginosa, BSI: Bloodstream infection, ICU: Intensive care unit.

**Table 3:** Univariate Cox model for the 30-day case-fatality rate in patients with PA bacteremic pneumonia.

Variables	Alive *n* = 132	Dead *n* = 162	HR	CI 95%	*p*-Value
Age (y), mean (SD)	61.6 (14.8)	61.1 (13.6)	1.00	0.99–1.01	0.781
Gender (female)	41 (31.1)	51 (31.5)	1.13	0.81–1.58	0.534
Acute leukemia	39 (29.5)	56 (34.6)	1.12	0.81–1.55	0.448
Refractory disease	48 (36.4)	71 (43.8)	1.29	0.95–1.76	0.122
HSCT	20 (15.2)	35 (21.6)	1.17	0.81–1.71	0.344
GVHD	7 (43.8)	14 (48.3)	1.09	0.53–2.27	0.806
Comorbidities ^a^	77 (62.1)	95 (60.1)	0.97	0.71–1.34	0.810
BSI acquisition (hospital-acquired)	58 (43.9)	88 (54.3)	1.18	0.87–1.61	0.223
Prior corticosteroid treatment (1 month)	71 (53.8)	94 (58.8)	1.13	0.83–1.55	0.417
Severe neutropenia	68 (54)	96 (59.6)	1.26	0.92–1.73	0.165
Septic shock	34 (26)	107 (66)	3.56	2.56–4.94	<0.001
Multidrug-resistant strain	11 (8.3)	41 (25.3)	2.05	1.43–2.93	<0.001
Inappropriate empirical antibiotic treatment	21 (15.9)	47 (29.0)	1.57	1.21–2.21	0.009
**Empirical treatment adequacy**					0.016
Inappropriate empirical antibiotic treatment	21 (15.9)	47 (29)	Ref	Ref	Ref
Appropriate empirical treatment (monotherapy)	65 (49.2)	82 (50.6)	0.75	0.52–1.07	0.115
Appropriate empirical treatment (combination treatment)	46 (34.8)	33 (20.4)	0.46	0.29–0.72	0.001

Abbreviations: PA: Pseudomonas aeruginosa; BSI: bloodstream infection; HSCT: Hematopoietic stem cell transplant; GVHD: Graft versus host disease; HR: Hazard Ratio; CI: Confidence Interval. ^a^ Comorbidities are defined as the presence of one or more of the following diseases: COPD, heart or hepatic disease, diabetes mellitus, renal failure, and cerebrovascular disease.

**Table 4:** Multivariate Cox model for the 30-day case-fatality rate in patients with PA bacteremic pneumonia.

Predictors	30-Day Case-Fatality Rate			
	aHR	Std. Error	*CI 95%*	*p*-Value
Age	1.00	0.01	0.99–1.01	0.616
Gender	1.17	0.20	0.83–1.64	0.376
Inappropriate empirical antibiotic treatment	1.44	0.26	1.01–2.03	0.042
**Septic shock**				
Group 1 (0–48 h from BSI onset)	6.53	1.87	3.73–11.43	<0.001
Group 2 (48 h–10 days from BSI onset)	2.89	0.22	1.66–5.05	<0.001
Group 3 (10 days–30 days from BSI onset)	1.41	0.58	0.63–3.14	0.400

Abbreviations: PA: Pseudomonas aeruginosa; BSI: bloodstream infection; aHR: adjusted Hazard Ratio, CI: Confidence Interval.

## Data Availability

Not applicable.

## Supplementary Materials

The following supporting information can be downloaded at: https://www.mdpi.com/article/10.3390/microorganisms10040733/s1, detailed list of the number of participating centers in each country and Table S1. Clinical and microbiological characteristics of patients with *Pseudomonas aeruginosa* bacteremic pneumonia.
