# Supplementary material for: Effect of Combination Antibiotic Empirical Therapy on Mortality in Neutropenic Cancer Patients with Pseudomonas aeruginosa Pneumonia
Source: Microorganisms. 2022 Mar 29;10(4):733. doi: 10.3390/microorganisms10040733 (PMC9027680; doi:10.3390/microorganisms10040733)
Supplement: Supplementary file 1 [file microorganisms-10-00733-s001.zip › microorganisms-1632614-supplementary.pdf]

Supplementary Material

Participating centers:

- Spain $n = 14$
- Turkey $n = 4$
- Brazil $n = 3$
- Italy $n = 3$
- Argentina $n = 2$
- Germany $n = 2$
- Chile $n = 1$
- Colombia $n = 1$
- Lebanon $n = 1$
- Slovakia $n = 1$
- Switzerland $n = 1$
- United Kingdom $n = 1$

Table S1. Clinical and microbiological characteristics of patients with *Pseudomonas aeruginosa* bacteremic pneumonia.

	PA pneumonia *n=294 (%)*	*PA BSI* *n= 723 (%)*	*p*
**Age (y), median (IQR)**	64 (55-71)	58 (47-68)	<0.001
**Gender (Female)**	92 (31.3)	284 (39.3)	0.02
**Type of underlying disease**			
**- Hematological disease**	201 (68.4)	567 (78.4)	0.005
• *Acute leukemia (AML, ALL)*	95 (32.3)	261 (36.1)	
• *Lymphoma (NHL, HL)*	56 (19)	185 (25.6)	
• *CLL*	20 (6.8)	16 (2.2)	
• *MM*	11 (3.7)	51 (7)	
• *MDS*	8 (2.7)	19 (2.6)	
• *Aplastic anemia*	5 (1.7)	13 (1.8)	
• *Other*	6 (2)	22 (3)	
**- Solid tumor**	93 (31.6)	156 (21.6)	<0.001
• *Lung cancer*	54 (18.4)	32 (4.4)	
• *Head and neck*	14 (4.8)	9 (1.2)	
• *Breast cancer*	6 (2)	19 (2.6)	
• *Urinary tumor*	4 (1.4)	21 (2.9)	
• *Upper GIT*	4 (1.4)	13 (7.8)	
• *Sarcoma*	2 (0.7)	15 (2.1)	
• *Other*	9 (3.1)	47 (6.5)	
**Treatment response**			
• *Refractory disease*	119 (40.5)	264 (36.5)	0.3
**HSCT**	55 (18.7)	192 (26.6)	0.01
**Type of HSCT**			0.1
• **Allo-HSCT**	40 (72.7)	114 (59.4)	
• **Auto-HSCT**	15 (27.3)	78 (40.6)	
**GVHD**	21 (7.1)	44 (6.1)	0.3
**Comorbidities** [a]	172 (58.5)	304 (42)	< 0.001
**BSI acquisition**			0.03
- Hospital-acquired BSI	146 (49.7)	413 (57.1)	
- Health-care related BSI	89 (30.3)	210 (29)	
- Community-acquired BSI	59 (20.1)	100 (13.8)	
**Prior corticosteroid treatment (1 month)**	165 (56.1)	362 (50)	0.16
**Severe mucositis**	32 (10.9)	97 (13.4)	0.32
**Septic shock at presentation**	141 (48)	192 (26.6)	<0.001
**Gangrenous ecthyma**	9 (3.1)	38 (5.3)	0.19
**Severe neutropenia (NAC < 100)**	164 (55.8)	441 (61)	0.11
**Source of BSI**			
- Pneumonia	294 (100)	0 (0)	
- Endogenous/Unknown	0	392 (54.2)	
- Catheter infection	0	87 (12)	
- Urinary tract infection	0	36 (5)	
- Skin and soft tissue infection	0	61 (8.4)	
- Neutropenic enterocolitis	0	46 (6.4)	
- Perianal infection	0	29 (4)	
- Mucositis	0	14 (1.9)	
- Other abdominal (cholangitis, peritonitis)	0	45 (6.2)	
- Other sources	0	13 (1.8)	

**Microbiological characteristics**			
Multidrug-resistant PA	52 (17.7)	108 (14.9)	0.33
Quinolone-resistant	100 (34)	203 (28.1)	0.07
Carbapenem-resistant	90 (30.6)	209 (28.9)	0.7

Qualitative data are expressed as numbers (%), unless otherwise indicated. Quantitative data are expressed as means ± standard deviation (SD) or median and interquartile range (IQR, 25th-75th percentiles), as appropriate.

Abbreviations: PA: *Pseudomonas aeruginosa*; BSI: bloodstream infection; AML: Acute myeloid leukemia; ALL: Acute lymphoblastic leukemia; HL: Hodgkin lymphoma; NHL: Non Hodgkin lymphoma; CLL: Chronic lymphocytic leukemia; MM: Multiple myeloma; MDS: myelodisplastic syndrome; GIT: Gastrointestinal tumor; HSCT: Hematopoietic stem cell transplant; GVHD: Graft versus host disease; NAC: absolute neutrophil count.

[a]Comorbidities are defined as the presence of one or more of the following diseases: COPD, heart or hepatic disease, diabetes mellitus, renal failure, and cerebrovascular disease
